# Properties of the surface electromyogram following traumatic spinal cord injury: a scoping review

**DOI:** 10.1186/s12984-021-00888-2

**Published:** 2021-06-29

**Authors:** Gustavo Balbinot, Guijin Li, Matheus Joner Wiest, Maureen Pakosh, Julio Cesar Furlan, Sukhvinder Kalsi-Ryan, Jose Zariffa

**Affiliations:** 1 KITE-Toronto Rehabilitation Institute, University Health Network, Toronto, ON M5G 2A2 Canada; 2 Institute of Biomedical Engineering, University of Toronto, Toronto, Canada; 3 Library & Information Services, Toronto Rehabilitation Institute, University Health Network, Toronto, Canada; 4 Rehabilitation Sciences Institute, University of Toronto, Toronto, Canada; 5 Department of Medicine, Division of Physical Medicine and Rehabilitation, University of Toronto, Toronto, Canada; 6 Division of Physical Medicine and Rehabilitation, Toronto Rehabilitation Institute, University Health Network, Toronto, Canada; 7 Institute of Medical Sciences, University of Toronto, Toronto, Canada; 8 Department of Physical Therapy, University of Toronto, Toronto, Canada; 9 Edward S. Rogers Sr. Department of Electrical and Computer Engineering, University of Toronto, Toronto, Canada

**Keywords:** Surface electromyography, Spinal cord injuries, Scoping review, Electrophysiology

## Abstract

Traumatic spinal cord injury (SCI) disrupts spinal and supraspinal pathways, and this process is reflected in changes in surface electromyography (sEMG). sEMG is an informative complement to current clinical testing and can capture the residual motor command in great detail—including in muscles below the level of injury with seemingly absent motor activities. In this comprehensive review, we sought to describe how the sEMG properties are changed after SCI. We conducted a systematic literature search followed by a narrative review focusing on sEMG analysis techniques and signal properties post-SCI. We found that early reports were mostly focused on the qualitative analysis of sEMG patterns and evolved to semi-quantitative scores and a more detailed amplitude-based quantification. Nonetheless, recent studies are still constrained to an amplitude-based analysis of the sEMG, and there are opportunities to more broadly characterize the time- and frequency-domain properties of the signal as well as to take fuller advantage of high-density EMG techniques. We recommend the incorporation of a broader range of signal properties into the neurophysiological assessment post-SCI and the development of a greater understanding of the relation between these sEMG properties and underlying physiology. Enhanced sEMG analysis could contribute to a more complete description of the effects of SCI on upper and lower motor neuron function and their interactions, and also assist in understanding the mechanisms of change following neuromodulation or exercise therapy.

**Supplementary Information:**

The online version contains supplementary material available at 10.1186/s12984-021-00888-2.

## Introduction

Traumatic spinal cord injury (SCI) may lead to severe sensorimotor dysfunction depending on the level and severity of injury. Changes in motor properties include muscle atrophy, muscle fiber type transformation, and increased passive stiffness in muscles and tendons [1], resulting in significant reductions in muscle strength, coordination [2], and functionality [3]. Symptoms of motor impairment are detected clinically by assessing the residual strength, sensibility and/or muscle activation of distinct muscle groups affected by the lesion using, for example, manual muscle testing (MMT). Clinical motor assessments such as the International Standards for Neurological Classification of Spinal Cord Injury (ISNCSCI) and the Graded Redefined Assessment of Strength, Sensibility, and Prehension (GRASSP) provide valuable information regarding strength and function [4–6]. This information can be further supplemented by electrophysiological approaches, among which a non-invasive assessment using the surface electromyogram (sEMG) has a number of advantages [7].

**Fig. 1:**
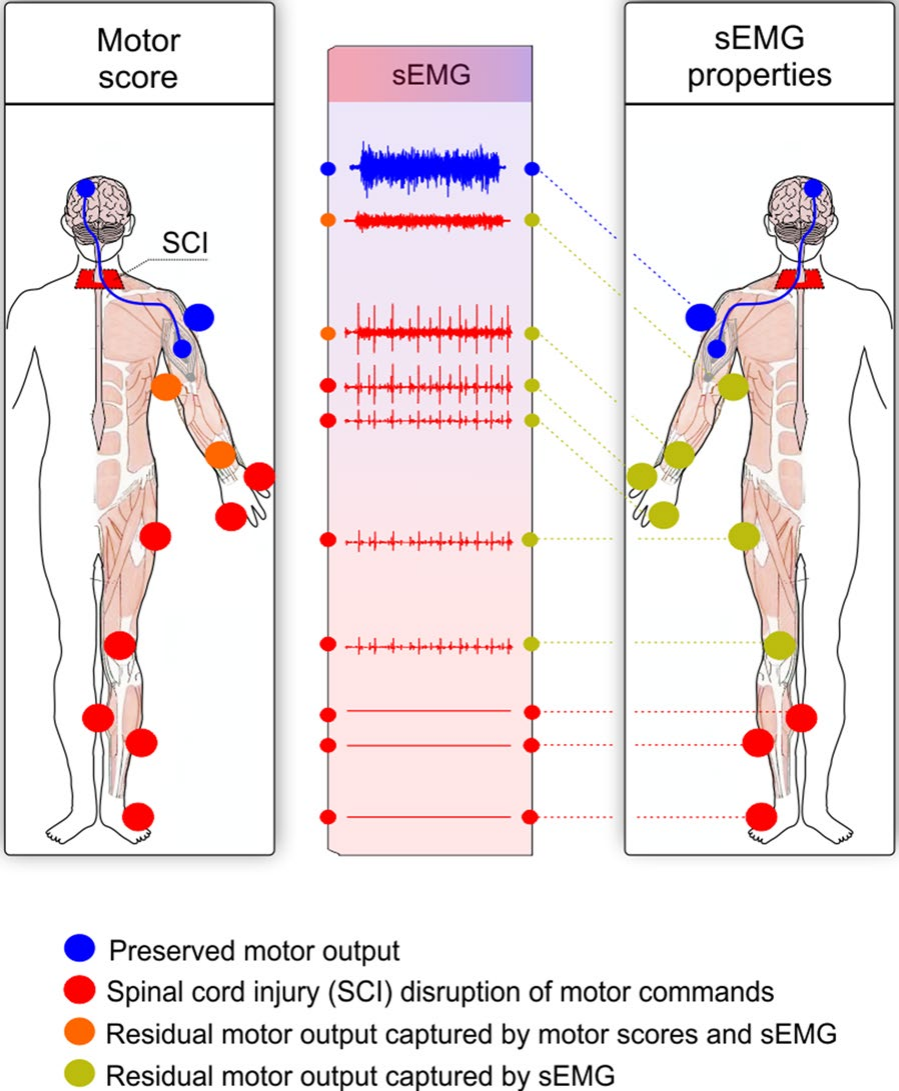
The surface electromyography (sEMG) is sensitive to detect residual motor commands from muscles with motor scores of zero. Spinal cord injury (SCI, red) disrupts motor commands from the brain (blue), hampering the motor output. The figure shows a hypothetical example using the main muscles assessed in the International Standards for Neurological Classification of Spinal Cord Injury (ISNCSCI). The motor output is commonly measured using motor scores (blue, orange or red dots in the left figure for intact, impaired or absent output, respectively). The sEMG assessment is able to capture the residual motor output in greater detail compared to motor scores because muscles with no motor scores can still display sEMG activity (blue, yellow or red dots in the right-side figure for normal, altered or absent sEMG, respectively). Symmetrical impairment has been assumed for ease of visualization

sEMG has been suggested to be a good marker for muscle health and function [8]. Importantly, sEMG amplitude highly correlates with strength and recovery, and can detect muscle activity in patients with no visible movement below the spinal injury level [9–11] (Fig. 1). sEMG assessments are not constrained by ceiling effects, with high variability of sEMG amplitude for example reported in individuals whose muscles were at a given ‘ceiling’ motor score of 5/5 (using MMT) [12]. Furthermore, sEMG can be beneficial to investigate muscles whose strength is difficult to measure, such as at thoracic levels. sEMG allows us to assess in high resolution the activity of several muscles at the same time during complex motor tasks, including activities of daily living, gait or reaching to grasping movements. It further allows the exploration of neuromuscular properties at rest or under passive movements, the residual control of volitional activity by the motor cortex, and the spontaneous or reflex activity intrinsic to the spinal cord [13]. Given the variety and depth of information that can be obtained from sEMG, an accurate interpretation of the signal properties after SCI is needed.

In this context, characterizing the impact of the SCI itself on sEMG is of interest for multiple reasons. First, injury-related changes and spontaneous recovery in the sEMG must be understood in order to isolate the impact of interventions. Second, neural interfaces that rely on sEMG require a good understanding of the expected signal properties. Third, a thorough understanding of what information can and cannot be obtained from non-invasive methods is essential to promoting the translation of electrophysiological techniques into routine clinical use. How are the sEMG properties changed after SCI? The answer to this question is not trivial, especially given that the sEMG signal reflects the net output of complex interactions between intrinsic spinal cord circuits, motor axon properties, and muscular mechanisms. sEMG is a well-established methodology to explore muscle activity after SCI; however, there is no consensus on data analysis and reporting strategies. The most common reporting methodology utilizes the signal amplitude, typically representing the average or maximal value recorded at a given time or window after some smoothing and normalization steps. However, the raw sEMG signal has the potential to be analyzed and expressed in many ways using temporal, spectral, and spatial techniques. A broad understanding of sEMG changes after SCI is of great interest in order to fully realize the potential of this measurement modality to characterize neurorecovery and support the development of neurorehabilitation technologies [14–16].

This scoping review aims to summarize and critically appraise the existing literature on how SCI can alter the sEMG properties. We distilled the large SCI literature reporting sEMG properties during residual volitional movements or abnormal spontaneous activity. These two domains are clinically significant given that neurorehabilitative interventions after SCI are thought to increase volitional control and reduce spontaneous activity. Accordingly, our results are structured around the different properties of the sEMG that can be expected to change after SCI. Insights into the neurophysiological basis of these phenomena are discussed and a new evidence-based perspective on the use of sEMG after SCI is provided.

## Methods

### Registry of Systematic Review Protocol

This is a scoping review using a systematic search (hybrid review). The systematic search was registered within the International Prospective Register of Systematic Reviews (https://www.crd.york.ac.uk/prospero/#aboutregpage; PROSPERO; Registration number CRD42020159040) and the Open Science Framework (https://osf.io/cg6yu/). Subsequently, a systematic review of the literature was conducted according to the checklist for the Preferred Reporting Items for Systematic reviews and Meta-Analyses extension for Scoping Reviews (PRISMA-ScR) Checklist [17].

### Information sources

Seven electronic databases were searched to avoid a biased literature sample: Medline (Ovid; 1510 entries), Cochrane Central Register of Controlled Trials (116 entries), Cochrane Database of Systematic Reviews (17 entries), Embase (1680 entries), Emcare (555 entries), Cumulative Index to Nursing & Allied Health Literature (CINAHL; 295 entries), and PubMed (non-Medline; 157 entries). The searches were originally performed on December 23rd, 2019 with no time limitations and re-run on September 22nd, 2020. No limits were applied for language to avoid excluding references not assigned to a language. References were also searched manually by reviewing reference lists of the included studies. Refer to Additional file 1: Table S1 for the Medline search strategy; similar strategies were used for the other six databases.

### Eligibility criteria

To be included, studies had to: (1) report sEMG properties in SCI participants (≥ 4 SCI individuals); (2) include participants with traumatic SCI (> 50% of the total SCI sample); and (3) be written in English. Regarding (1), we defined the sample size threshold a priori to accommodate the low prevalence characteristic of SCI while balancing the need to select studies that contain generalizable information. In (2), despite the obvious differences in the time profile of disease onset in traumatic and non-traumatic (e.g., degenerative cervical myelopathy) SCI, it is thought that both lead to similar white matter degeneration [18]. Further studies are needed to understand the existence of subtle white matter degeneration between these conditions [18]. To the best of our knowledge, there is no evidence of changes in sEMG properties between these conditions. However, we decided to take a conservative approach and excluded articles where most participants were impaired due to a non-traumatic SCI (one article). Conference abstracts were excluded due to the lack of full methods and complete data sets. Theses and dissertations were also excluded because it is unclear whether they were peer-reviewed. Case studies or case reports with less than four participants were excluded because of the low sample size and statistical heterogeneity. Studies on respiratory muscles (e.g., diaphragm), sphincter, pelvic floor, or smooth muscles were not included given that some of these muscles are deep (better assessed using intramuscular EMG), display distinct physiological properties (e.g., smooth muscles), or rely to a large extent on rhythmic rather than volitional movement. Studies exploring intramuscular EMG were excluded. Finally, studies aiming at assessing the effects of treatments or interventions such as pharmacotherapy and neurostimulation were also excluded, as our focus was on understanding the impact of the SCI itself on the sEMG.

### Search strategy

A PICO model (Problem/Patient/Population, Intervention/Indicator, Comparison, and Outcome) was used to build search criteria for the electronic databases. The PICO consisted of Population: “Spinal Cord Injury”, Intervention/Identifier: “sEMG”, and Outcome of interest: “Muscle/Motor Response”. Valid subject headings as appropriate for each database were utilized in the search strategies, as were free-text terms relevant to each topical concept.

### Study selection

Duplicate references were removed manually in addition to using Covidence (Melbourne, Australia) and Mendeley software (Mendeley Inc., New York, NY, USA), and manually. Two authors (GB and GL) independently screened titles and abstracts to determine initial eligibility. Eligible references were included for full-text screening. Conflicts were resolved by a third reviewer (MJW).

### Data extraction

Data extraction from each full-text article was completed by the first author (GB) using a personalized spreadsheet, which was pilot-tested and refined using 5% of the references that passed the full-text screening. Extracted data included: (1) study identification information (author and year); (2) study design; (3) participant demographics: level of injury, American Spinal Injury Association Impairment Scale (AIS), time post-injury, sex, and age; (4) sample size; (5) the motives for using sEMG; (6) muscles evaluated; (7) sEMG equipment and electrodes; (8) electrodes placement and reliability information; (9) sEMG data analysis and reporting strategy; (10) main sEMG findings: means, standard deviations and p values for relevant outcome measures; (11) measures other than sEMG. If insufficient data were reported, the authors were contacted by email.

### sEMG scores

Following the literature search and selection, a data score was assigned by the first author (GB) as follows to sEMG methodology description was rated as present or not using the following criteria: sEMG equipment/electrode and amplification/filtering description was considered present (yes) if the type and settings of equipment were described in sufficient details (e.g., yes: “surface Ag/AgCl electrodes with 2 cm diameter” or no: “surface electrodes”). Similarly, the electrode placement was considered as present if it was described in sufficient detail (e.g., a description of “electrodes were placed at the T-10 vertebral level, 2 cm lateral from midline” in contrast to “placed on the muscle belly”) or based on established references (e.g., yes: SENIAM recommendations for EMG recording procedures). We also rated the sEMG findings employed in the studies using a 3-point scale: qualitative (0; based on sEMG visuals/graphs), semi-quantitative scores (1; e.g., scale from 0 to 5) or quantitative (2).

## Results

### Overview

**Fig. 2:**
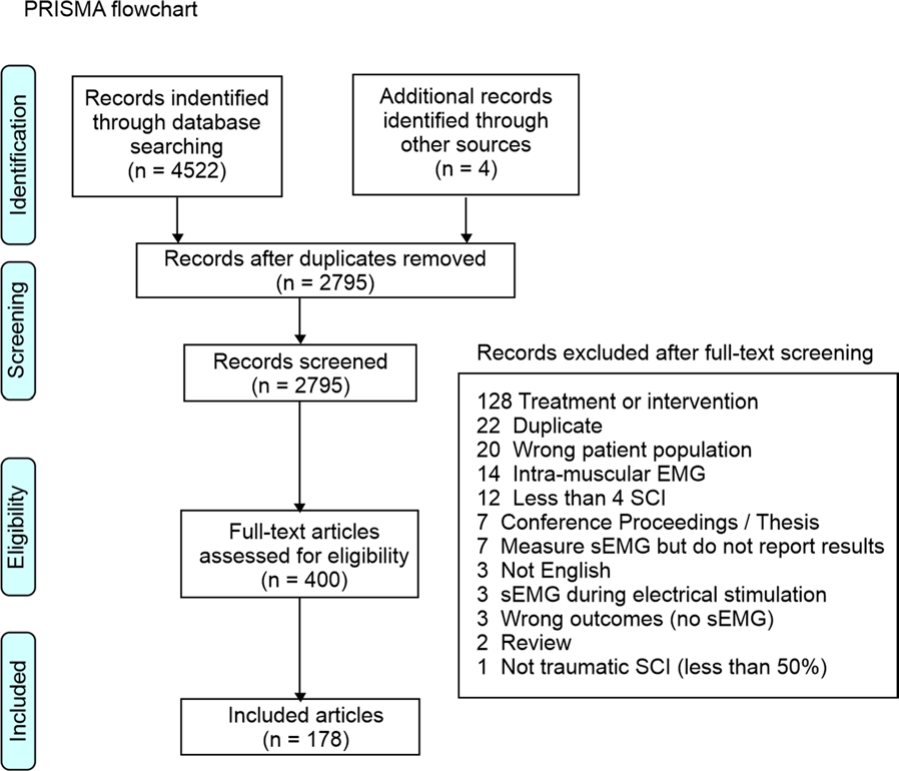
PRISMA flowchart. *PRISMA* Preferred Reporting Items for Systematic reviews and Meta-Analyses

The PRISMA flowchart is described in Fig. 2. Of 4522 references initially captured in the primary search, 175 references were selected and included in the scoping review. Properties of the sEMG following SCI are described considering volitional effort to reflect supraspinal control of movement or the rest/spontaneous activity to reflect pathologic activation intrinsic to the spinal cord. These two domains are intertwined and dependent on dysfunction patterns of upper motor neuron (UMN) and lower motor neuron (LMN). As such, integrative considerations are discussed throughout.

#### Injury and muscle characteristic and participant demographics

**Fig. 3:**
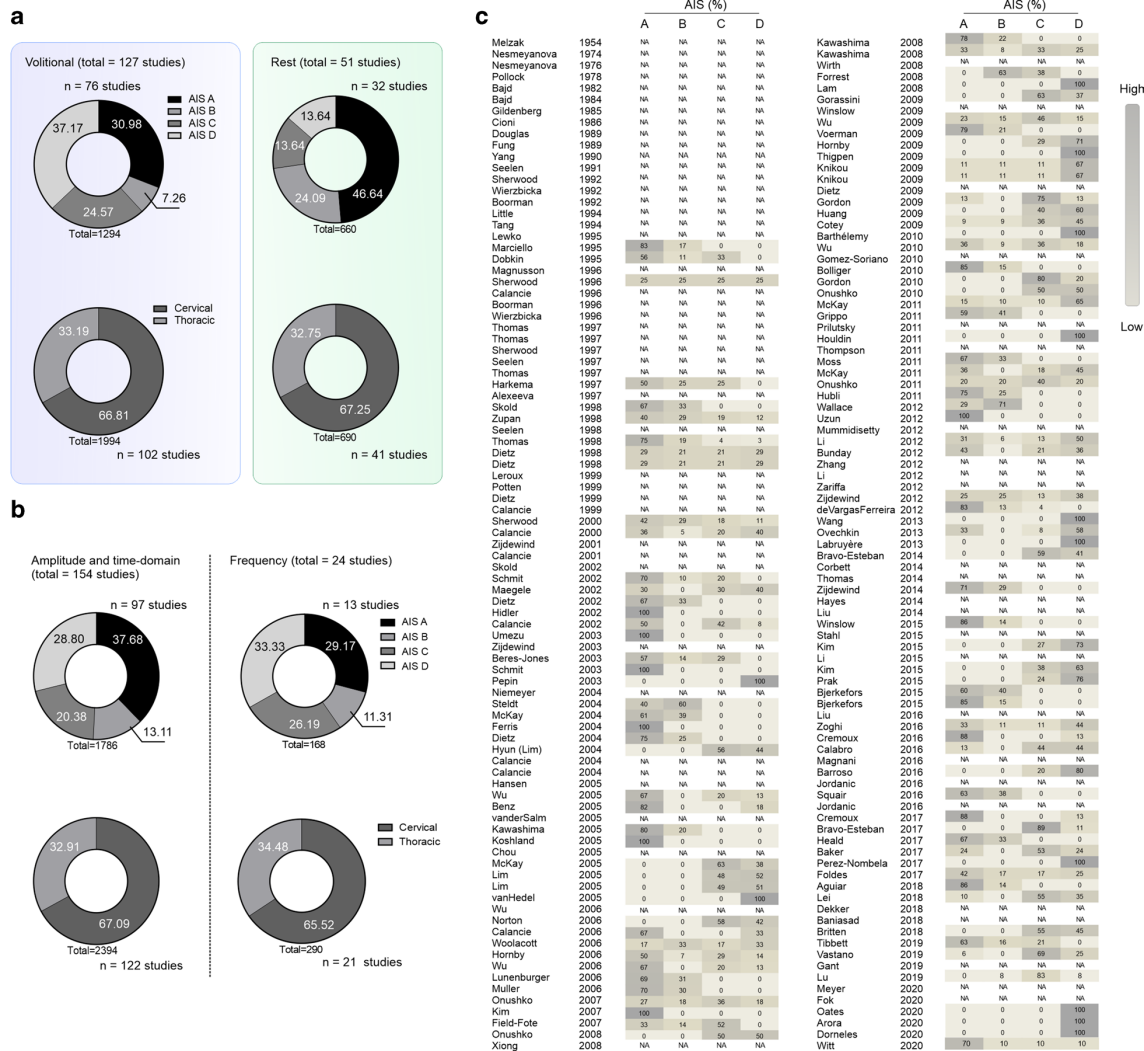
Surface electromyography (sEMG) and American Spinal Injury Association Impairment Scale (AIS) classification. **a** AIS classification and level of injury of participants assessed using sEMG measured at volitional effort or rest. **b** AIS classification and level of injury of participants in studies reporting sEMG properties in the amplitude and frequency domains. **c** Historical perspective of AIS distributions across studies using sEMG assessments. *sEMG* surface EMG, *SCI* spinal cord injury, *AIS* American Spinal Injury Association Impairment Scale, *NA* not available (i.e. not described or described in insufficient details)

Most of the references identified used the ASIA Impairment Scale (AIS) component of the ISNCSCI to describe their study sample (108 of 178 studies) when measuring sEMG properties at rest (32 studies) or during volitional effort (76 studies). Studies assessing sEMG at rest included more AIS A and B participants (≈ 71% of studies), while studies assessing volitional control included mostly AIS C and D (≈ 62% of studies). Most of the studies (≈ 67%) were conducted in cervical lesions, indicating sensorimotor impairments in the upper and lower limbs (Fig. 3a, b).

For historical reasons, studies before 1995 did not include AIS information (Fig. 3c). However, some recent work also describes participant injury state only with complete or incomplete or as a mix of gradings, without clear descriptions of impairments.

**Fig. 4:**
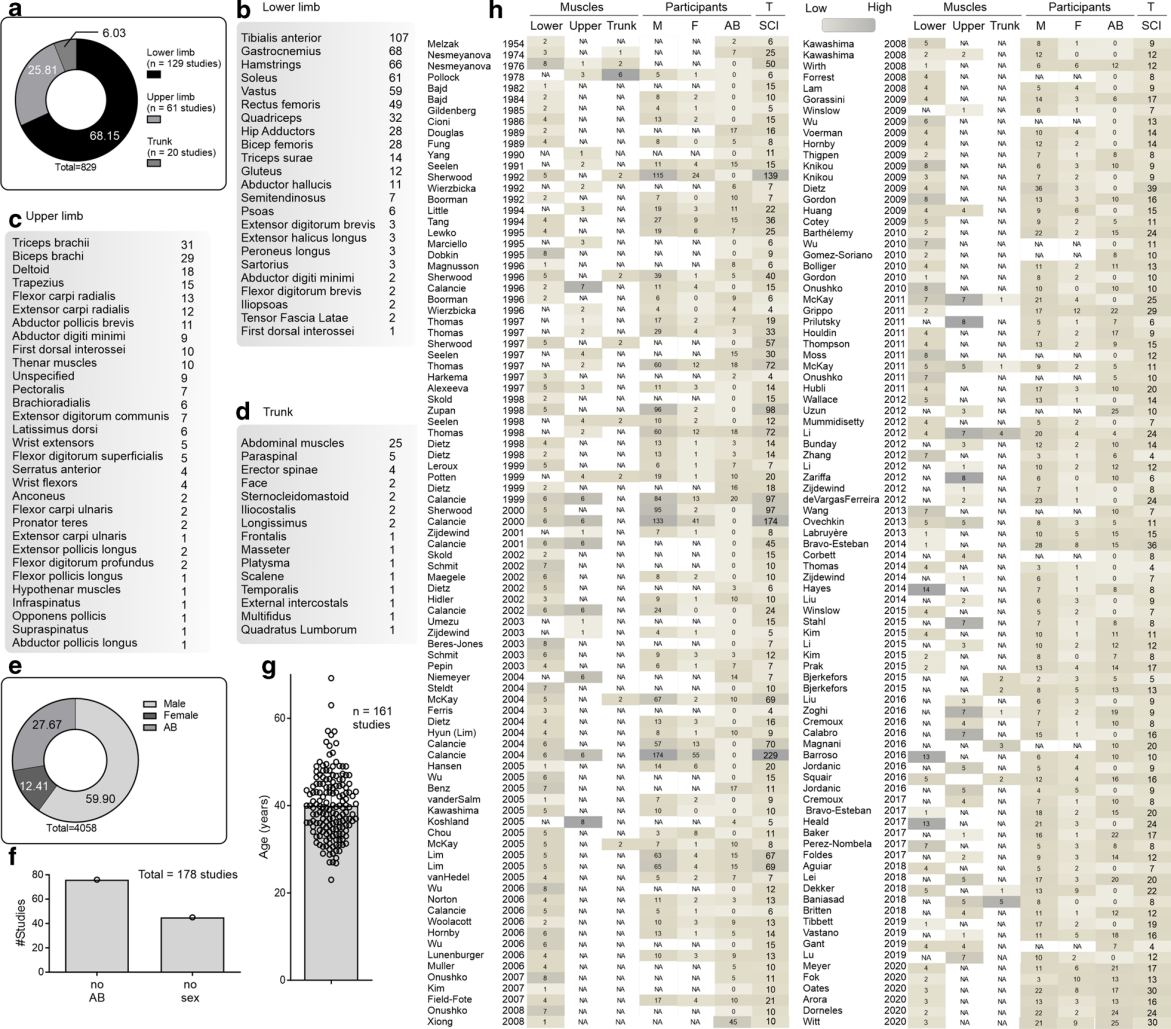
Characteristics of muscles and spinal cord injured (SCI) participants. **a** The proportion of upper limb, lower limb, and trunk muscles assessed using sEMG. **b** The number of studies assessing individual lower limb, **c** upper limb, and **d** trunk muscles. **e** The proportion of male, female, and control (able-bodied, AB) participants in sEMG assessments. **f** The number of studies not reporting the use of controls (AB) or sex. **g** The age of SCI participants at the time of the sEMG assessment. **h** Historical perspective of the muscles and participants studied. *AB* able-bodied, *M* male, *F* female, *T* total, *SCI* spinal cord injury

Many of the studies assessed the properties of sEMG in lower limb muscles (≈ 68% of studies), a substantial amount in upper limbs muscles (≈ 26% of studies), and only a few studies in the trunk/head muscles (≈ 6% of studies) (Fig. 4a). Tibialis anterior and gastrocnemius were the most studied among lower limb muscles, triceps and biceps brachii among upper limb muscles, and abdominal muscles for the trunk/head (Fig. 4b–d). Sixty-one percent of the extracted data came from male participants with SCI, 29% from able-bodied (AB) control participants, and only 13% from female participants with SCI (Fig. 4e). Seventy-six and 45 studies of 178 did not use an AB control group or report sex, respectively (Fig. 4f). The average age of SCI participants was 40 years (range 23–69 years; SD ± 7.5 years; Fig. 4g).

The interest in lower limb muscles, the predominance of males, and lack of AB control groups (Fig. 4h) can be explained from historical perspectives, but some recent studies are still failing to report sex or gender.

**Fig. 5:**
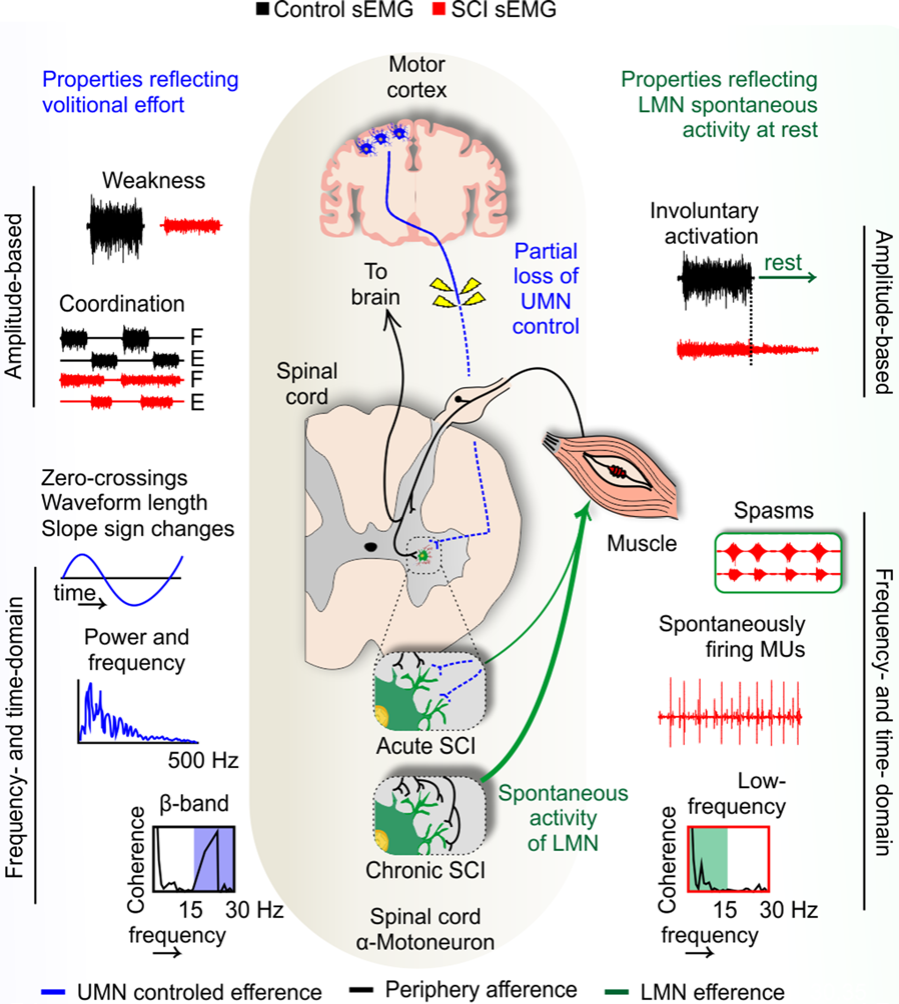
Surface electromyography (sEMG) properties following spinal cord injury (SCI). Left: Changes in sEMG properties may be related to weak cortical control (hashed blue lines) but also to aberrant plasticity within the intrinsic spinal cord circuitry (green lines). For example, the lack of UMN control may be reflected in reduced muscle strength and coordination to control volitional muscle activity, reflected in amplitude- and frequency-based sEMG properties [e.g., amplitude: root mean square (RMS), peak; frequency: median frequency (MDF), power spectrum density (PSD), intra- or intermuscular coherence at the β-band]. Right: The lack of UMN efferents to the spinal cord induces sprouting within the spinal cord circuitry. Given the intact afferents arriving from the periphery and/or pathways within the spinal cord, the intrinsic spinal cord circuitry is prone to hyperexcitability. This is reflected in the amplitude-based sEMG properties as an involuntary activation, many times propagating to several muscle groups in the form of tonic, clonic, or unit spams. These forms of spontaneous activity, thought to involve intrinsic spinal cord circuitry, are detected at rest and present unique frequency-based sEMG features such as low-frequency muscular coherence. *SCI* spinal cord injury, *sEMG* superficial electromyography, *UMN* upper motor neuron, *F* flexors, *E* extensors, *Hz* hertz, *LMN* lower motor neuron

The following sections summarize the currently available evidence about alterations in the characteristics of the sEMG signal after SCI. The focus of these investigations is summarized in Fig. 5.

### Properties reflecting volitional control

#### Amplitude-based sEMG during volitional efforts

Using qualitative sEMG graph analysis, early investigations explored the use of volitional muscle activation during postural movements, which described postural patterns and the tendency for clonus of hip flexors/extensors during the act of rising [19], as well as the emergence of abnormal movement synergies following SCI [20, 21].

With the progression of sEMG technology, researchers were able to assess multiple muscle groups simultaneously with higher resolution. This facilitated the use of sEMG as a means to control assistive devices, as different sites for myoelectric control in individuals with high-level quadriplegia were tested using integrated sEMG amplitude to detect the amount of volitional control over trunk/head muscles [22]. It was evident to these pioneers that sEMG during volitional effort had the ability to detect motor control alterations and was applicable to assistive technology. Nonetheless, some of these early studies employed qualitative assessment of sEMG data (e.g., the amplitude and shape of the sEMG signal) to make inferences about the neurophysiological and functional status of individuals with SCI.

##### Muscle weakness and spontaneous recovery in acute and sub-acute SCI

**Fig. 6:**
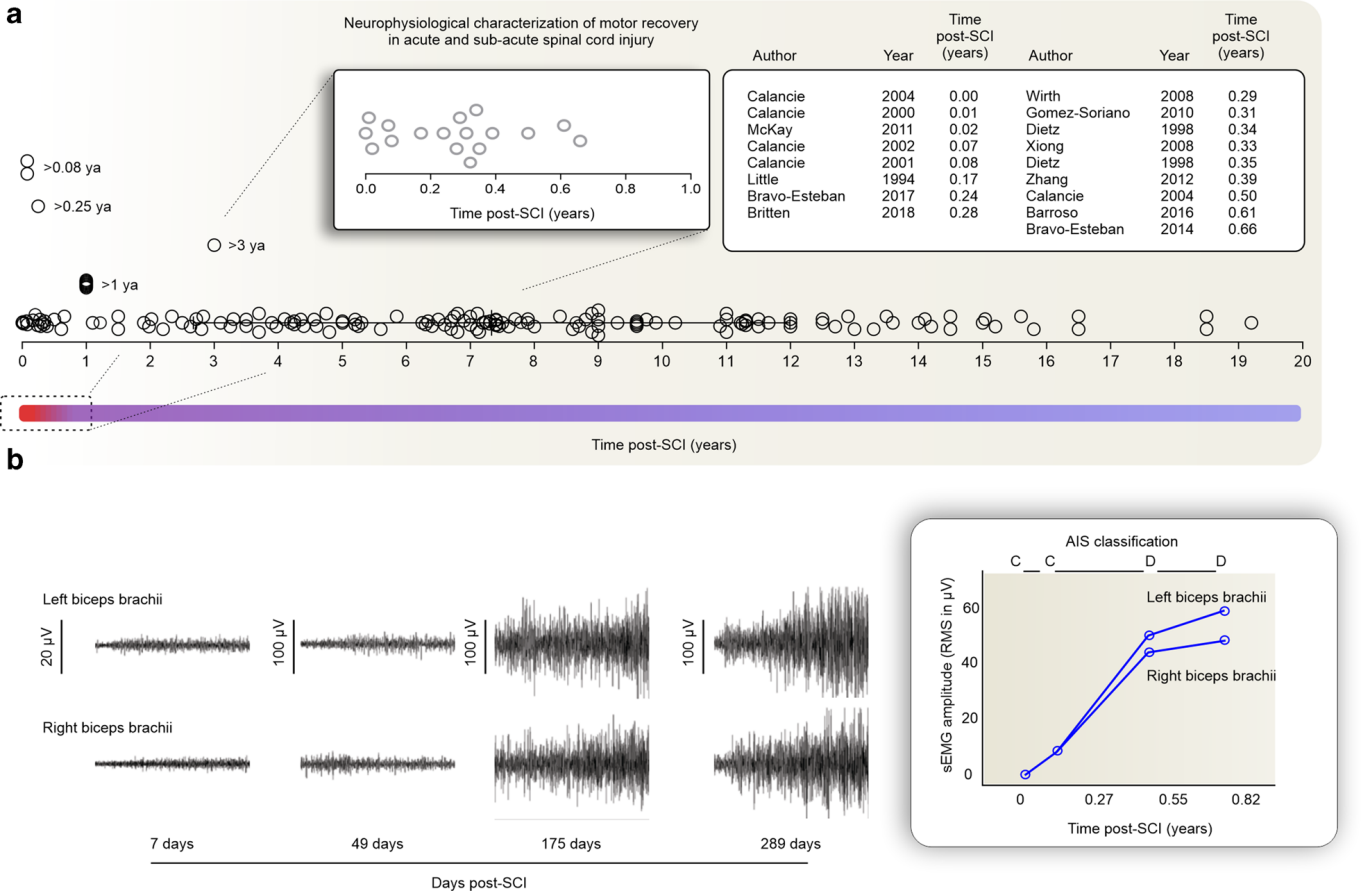
Neurophysiological characterization of spontaneous recovery. **a** 18 sEMG studies were conducted in the acute/sub-acute phase of SCI, while the majority of studies were conducted during the chronic phase of SCI (mean = 7.33 years). **b** Spontaneous recovery of sEMG activity from a representative participant. Left: elbow flexor muscles after SCI. Right: averaged response from three 3-s trials recorded from the left and right biceps brachii muscles at 7, 49, 175, and 279 days post-SCI [12] (adapted with permission). *ya* year, *SCI* spinal cord injury, *AIS* American Spinal Injury Association Impairment Scale, *RMS* root mean square

Of 178 studies, only 18 were conducted during the acute and sub-acute phases of SCI (< 1-year post-injury) (Fig. 6a). Muscle weakness, paralysis, and atrophy were reported following SCI [23, 24]. The mean integrated sEMG generated by individuals with SCI during maximum voluntary contractions (MVCs) were significantly less than those produced by controls, with 71% of muscles generating less than 10% of AB control sEMG [23]. Although individuals with SCI usually produced lower MVC forces, an orderly recruitment of the few units that remain under voluntary control could be demonstrated [25]. Also, in sub-acute SCI, the number of motor units recruited seem to increase with time post-injury—from 40 ± 33 to 116 ± 41 [26].

The disruption of UMN and LMN activity at specific spinal cord segments results in muscle-specific weakness, which relates to the level of spinal cord lesion and determines muscle activity following SCI [27]. UMN and LMN lesions, mixed weakness, and their dependence on the level of injury were described in detail using sEMG, including amplitude-based properties during voluntary movements [28] and the use of transcranial magnetic stimulation (TMS) [29]. These studies challenged the integrity of the central nervous system using artificial electric or magnetic stimulation in conjunction with volitional sEMG measurements. By stimulating the peripheral nerve an M-wave is generated on the muscle and thought to involve the activation of all motor units, regardless of number and size. Thus, the M-wave indicated the maximal recruitment and if divided by the sEMG RMS captured during MVC can indicate the extent of volitional activation of motor units in relation to the maximum (the M/RMS ratio). The M/RMS ratio would be 0 if no M-wave could be produced, indicating LMN lesion, but weak muscles with M-wave amplitudes in the normal range displayed additional sEMG characteristics suggesting UMN dysfunction. For example, muscles with large M/RMS ratio and slow maximum motor unit firing rates (measured using intramuscular EMG) denoted predominant UMN weakness and muscles that showed both very small M-responses and large M/RMS ratios were deemed to represent mixed UMN and LMN impairment [28]. A similar approach using TMS over the motor cortex led to similar findings. Muscles presenting a high semi-quantitative sEMG score at volitional effort also presented greater evoked responses, indicating a relation between residual UMN control and volitional sEMG [29].

The solid relation between muscle strength and sEMG stemmed from the seminal work of Calancie and colleagues and opened new avenues in the application of sEMG in understanding neural plasticity and movement recovery following SCI [30]. These studies showed a consistent relationship between sEMG and muscle force after SCI [11] and the ability to assess the recovery of voluntary movement after acute SCI [10]. Spontaneous recovery of muscle force could be detected in the sEMG signal in great detail and scored using a simple 6-point scale to mimic the ISNCSCI muscle function grading (also from 0 to 5). Utilizing comparable sEMG scores between clinical and sEMG assessments increased the applicability of the sEMG in the clinical settings and the understanding of motor recovery following SCI. This included insights into the distribution and latency of muscle responses to TMS [31], abnormal interlimb responses [32], and the use of the sEMG interference pattern as a biomarker for monitoring lower limb recovery [33]. Most of these studies were performed in large cohorts of participants and provided valuable insights into the mechanisms underlying the restoration of axonal conduction in central motor pathways following SCI.

Dietz and colleagues integrated electrophysiological evaluations into their description of the spontaneous recovery of locomotor muscles over 26 weeks post-injury. sEMG amplitude and reflex activity were absent for up to 14 weeks post-SCI; following resolution of the spinal shock, tendon tap response reappeared, and the sEMG amplitude gradually increased over the next 4 weeks—and plateaued at around 22 weeks post-SCI [34, 35]. Further studies confirmed the gradual increase of muscle strength, gait and the motor evoked potentials amplitudes during the first 24 weeks following SCI. During this period, there was a persistent conduction delay within the corticospinal tract but the amplitude of the motor evoked potential increased at a stable level of background sEMG activity of 20% MVC (unchanged during the first 24 weeks following SCI) [36]. This gain in activation at stable background sEMG activity might indicate improved synchronization of the descending volley and/or responsiveness of motoneurons to supraspinal input with spontaneous recovery following SCI. In other words, since the corticospinal function did not show significant conduction improvement during motor recovery of acute SCI, it is likely that intrinsic spinal cord plasticity mechanisms play an important role in this process of spontaneous recovery [36]. The heterogeneity of the sEMG response following SCI was highlighted in further studies, and the spontaneous recovery found to be very individualized in terms of sEMG patterns and rate of change over time [37].

Finally, Sherwood and colleagues contributed to the understanding of muscle weakness and recovery following SCI through the introduction of the Brain Motor Control Assessment protocol. This comprehensive multichannel surface sEMG approach allowed a better characterization of motor control features in individuals with UMN dysfunction [38, 39]. The assessment consisted of a battery of tests initially using semi-quantitative features of the sEMG amplitude properties during relaxation (spontaneous firing), reinforcement (amplitude), voluntary movement (amplitude), tonic stretch response, phasic stretch response, presence of clonus, and response to vibration or plantar stimulation [38, 40]. Later, quantitative amplitude measurements of the RMS sEMG were added to this protocol and demonstrated strong between-day reliability [39] and relation to the Ashworth scale [41]. The Brain Motor Control Assessment protocol was further developed by using similarity indices, which compare the sEMG pattern between muscles from AB control and SCI participants [42]. This provided a more detailed description of the spontaneous recovery process [37, 43]. Spontaneous recovery was described in terms of significant increases in the ability to activate motor units on command, the rate at which those motor units were recruited, and the ability to appropriately organize the motor unit activation across the prime mover, antagonist, and distant muscles [37]. These findings were based on serial recordings made up to 33 weeks post-SCI and showed characteristics of the spontaneous recovery process reflected in the sEMG signal (Fig. 6a—inset). In some muscles, at first, only a few motor units fired; however, with recovery, an increase in amplitude was evident accompanied by a progressive decrease in the time from the onset of activity to the peak of activation. In addition to the use of similarity indices, the study by Mckay et al. [37] explored the ability to assess multiple muscle groups using sEMG. This highlighted that spontaneous recovery is not only more activation, but increased activation in the agonists accompanied by reduced co-activation of antagonist or distant muscles. An additional study assessed mixed cohorts of acute and chronic participants and showed the spontaneous recovery process from selected participants (Fig. 6b) [12].

Co-activation in sub-acute SCI was also studied longitudinally using amplitude-based RMS sEMG properties from 3 to 5 months post-SCI. The results suggested an unbalanced recovery of UMN control over muscles (co-activation) and the attenuation of the recovery process by the presence of lower limb hypertonia and involuntary muscle activity [44]. Muscle coordination and sEMG properties at rest will be discussed further in the following sections.

##### Volitional muscle activation in chronic SCI

Early reports of volitional muscle activation during the chronic phase of SCI (> 1-year post-injury) aimed at determining the optimal myoelectric control sites [22] and later the neurophysiological characteristics of UMN and LMN lesions [28]. sEMG was also used to investigate the feasibility of using muscles innervated below the injury level as command sources for a neuroprosthesis. The results indicated that although the preservation of a small number of axons alone may not be sufficient to produce functional movement, these signals are likely to be sufficient to control a motor neuroprosthesis [45]. sEMG was also able to capture small differences in well-recovered individuals with incomplete SCI at the chronic phase [46].

The ability of sEMG to capture residual impairments or muscle activity after SCI has been used to categorize muscles according to the presence or absence of detectable movement and determine the extent of preserved muscle activity [47, 48], including in abdominal muscles [49, 50]. Note that in muscles with absent volitional sEMG, an event-related desynchronization was evident when attempting to move, in contrast to SCI muscles with volitional sEMG and AB controls [51].

The Brain Motor Control Assessment introduced in “Muscle weakness and spontaneous recovery in acute and sub-acute SCI” has been applied in a series of studies on volitional control of muscle activity in chronic SCI. The Voluntary Response Index (the volitional component of the Brain Motor Control Assessment) was able to differentiate individuals with SCI from AB controls. In addition, the two components of the Voluntary Response Index, magnitude and similarity index, varied independently [52] and were related to preserved corticospinal connections [53]. This technique was also able to distinguish between the most and the least affected sides as well as between AIS D and AIS C individuals [54], and demonstrated good to excellent short- and intermediate-term reliability [55]. In summary, these results validate the sEMG-based Voluntary Response Index as an objective, quantitative, and repeatable laboratory measure of voluntary motor control disruption. The Brain Motor Control Assessment correlated with clinical scale scores acquired more than 48 days after injury; however, such correlations were not found for the first 19 days post-injury [56].

Muscle properties may also change in chronic SCI. The pattern of central motor drive of the plantar flexors indicated that individuals with SCI generated greater activation of the plantar flexors during eccentric MVCs compared with isometric or concentric MVCs, likely related to increased efficacy of muscle spindles Ia-α motoneuron transmission during lengthening contractions [57, 58]. Finally, the study of voluntary activation of weakened hand intrinsic muscles during sustained contractions indicated that impaired activation due to SCI was more important for explaining weakness compared to muscle atrophy, and greater central fatigue was likely offset by less peripheral fatigue as a result of lower muscle activation [59].

Novel technologies take advantage of high-density sEMG and wearable electrodes. High-density sEMG can provide more detailed information on patterns of muscle activation, which can also be summarized, for example, using the center of gravity [60, 61]. Wearable technology will soon provide the opportunity to characterize muscular activity under a greater range of sedentary and active conditions in the home environment [62, 63].

##### Motor control and coordination

sEMG has been used extensively to gain insights into motor control alterations after SCI. Lines of investigation have included abnormal synergies [20], completion time and accuracy of movements [64, 65], and patterns of co-activation [20, 66, 67]. Another noteworthy application is the study of compensatory movement strategies, which can be accompanied by the development of new muscle synergies [68–72]. Studies have commonly focused on these issues in the context of locomotion [3, 27, 34, 35, 73–98], while fewer studies focused on reach-to-grasp [99–107], and posture and balance [108–112].

While these research efforts have yielded considerable insights into neural control following SCI, most of them rely on similar methodologies to quantify the sEMG signal. The emphasis has been placed on timing information to describe patterns of activation, and normalized envelope amplitudes (a summary of the sEMG properties assessed in each study can be found in Table 1). A number of studies have then built on these representations to apply synergy extraction methods that can characterize changes in muscle coordination patterns [2, 103, 113–115]. Frequency-based descriptions of the sEMG have also been used in the context of motor control and coordination, and are summarized in the following section.

**Table 1.** Description of the main sEMG properties identified in SCI studies

sEMG properties	Description	References
Amplitude envelope	Commonly computed over a moving window using the root mean square, the average rectified signal, or the integrated rectified signal, or using low-pass filtering. The sEMG amplitude during voluntary contraction is decreased in muscles that are weakened as a result of reduced innervation. Conversely, sEMG may increase at rest after SCI in the event of spasms	Arora (2020); Baker (2017); Bjerkefors (2015a); Bjerkefors (2015b); Bolliger (2010); Boorman 1992); Cioni (1986); Corbett (2014); Cotey (2009); Cremoux (2016); deVargasFerreira (2012); Dietz 1998a); Dietz (1998b); Dietz (1999); Dietz (2002); Dietz (2004); Dietz (2009); Dorneles (2020); Ferris (2004); Field-Fote (2007); Fok (2020); Forrest (2008); Fung (1989); Gomez-Soriano (2010); Gordon (2009); Gordon (2010); Grippo (2011); Harkema (1997); Hornby (2006); Houldin (2011); Huang (2009); Hubli (2011); Hyun (2004); Jordanic (2016); Jordanic (2016); Kawashima (2005); Kawashima (2008a); Kawashima (2008b); Kim (2007); Kim (2015); Knikou (2009a); Knikou (2009b); Lam (2008); Li (2012a); Li (2012b); Lim and Sherwood (2005); Lim (2005); Little (1994); Liu (2014); Liu (2016); Lu (2019); Lunenburger (2006); Maegele (2002); Magnani (2016); Magnusson (1996); Marciello 1995); McKay (2004); McKay (2005); McKay (2011); Meyer (2020); Moss (2011); Muller (2006); Mummidisetty (2012); Niemeyer (2004); Oates (2020); Onushko (2007); Onushko (2008); Onushko 2010); Onushko (2011); Ovechkin (2013); Pepin (2003); Pollock (1978); Potten (1999); Prak (2015); Seelen (1991); Seelen (1997); Seelen (1998); Sherwood (1996); Sherwood (1997); Sherwood 2000); Skold (1998); Skold (2002); Squair (2016); Tang (1994); Thigpen (2009); Thomas (1997a); Thomas 1997b); Thomas (1998); Thomas (2014); Tibbett (2019); Uzun (2012); vanderSalm (2005); vanHedel (2005); Vastano (2019); Voerman (2009); Wallace (2012); Wierzbicka (1992); Wierzbicka 1996); Winslow (2015); Wirth (2008); Woolacott (2006); Wu (2005); Wu (2006); Wu (2009); Wu (2010); Zariffa (2012); Zijdewind (2003); Zijdewind (2014); Zoghi (2016); Zupan (1998).
Normalized amplitude	Amplitude expressed as a percentage of the value obtained at maximal voluntary contraction, at a point of interest in a time series, or using electrical stimulation. Normalization to MVC can be difficult to interpret after SCI because the maximal activation is itself affected by the injury. The M/RMS ratio has been used to differentiate UMN and LMN damage	Arora (2020); Bunday (2012); Calabro (2016); Cremoux (2016); Cremoux (2017); Dekker (2018); Hayes (2014); Dorneles (2020); Hornby (2009); Houldin (2011); Kim (2007); Kim (2015); Kim (2015); Lei (2018); Leroux (1999); Meyer (2020); Niemeyer (2004); Oates (2020); Prak (2015); Prilutsky (2011); Stahl (2015); Steldt (2004); Thigpen (2009); Thompson (2011); Tibbett (2019); Wallace (2012); Wang (2013); Wirth (2008); Wu (2005); Wu (2006); Wu (2010); Zijdewind (2003); Zijdewind (2012)
Peak amplitude	Maximal sEMG value in a time-series. Similar to the amplitude envelope, decreased values can be expected after SCI, depending on the pattern of injury	Britten (2018); Dobkin (1995); Gant (2019); Gorassini (2009); Heald (2017); Nesmeyanova (1974); Nesmeyanova (1976); Pepin (2003); Sherwood (1997); Skold (1998); Skold (2002); Thomas (1997); Wierzbicka (1996); Zijdewind (2012)
Onset/offset	Timing of sEMG activation defined when the signal amplitude is greater (onset) or lower (offset) than a pre-defined baseline threshold. Disruptions in the timing of muscle activation can be expected during complex movements (e.g. gait) after SCI, depending on the location and severity of the lesion	Aguiar (2018); Arora (2020); Bajd (1982); Bajd (1984); Baker (2017); Benz (2005); Beres-Jones (2003); Calancie (2006); Field-Fote (2007); Fok (2020); Forrest (2008); Fung (1989); Gorassini (2009); Harkema (1997); Hidler (2002); Kawashima (2005); Kawashima (2008); Kawashima (2008); Koshland (2005); Labruyère (2013); Leroux (1999); Liu (2016); Maegele (2002); Meyer (2020); Moss (2011); Mummidisetty (2012); Onushko (2010); Onushko (2011); Pepin (2003); Schmit (2002); Schmit (2003); Tang (1994); Tibbett (2019); vanderSalm (2005); Voerman (2009); Wallace (2012); Wierzbicka (1992); Wierzbicka (1996); Winslow (2009); Winslow (2015); Wu (2006); Wu (2009); Wu (2010)
Qualitative or semi-quantitative scores	Qualitative and semi-quantitative scores (e.g. present vs absent) based on the analysis of sEMG time-series. Semi-quantitative scoring criteria were established to approximate a manual muscle testing score, as follows: 0, no volitional recruitment or rate-modulation of spontaneously active motor units; 1, recruitment of 1 or 2 motor units, unsustained discharge; 2, recruitment of 1–3 motor units, sustained discharge, slow onset and/or offset; 3, recruitment of 3 motor units, but not enough to become indistinct audibly, sustained discharge, rapid onset and offset; 4, recruitment of multiple units, indistinguishable visually and audibly, rapid onset and offset, moderate amplitude at peak; and 5, recruitment of multiple units, indistinguishable visually and audibly, rapid onset and offset, high amplitude (> 0.1 mV) at peak	Alexeeva (1997); Calancie (1996); Calancie (1999); Calancie (2000); Calancie (2001); Calancie (2002); Calancie (2004); Calancie (2004); Chou (2005); Douglas (1989); Foldes (2017); Gildenberg (1985); Koshland (2005); Lewko (1995); Melzak (1954); Sherwood (1992)
Synergy analysis	Commonly obtained by using non-negative matrix factorization or principal component analysis to identify distinct patterns of activation across several muscles that can be combined to achieve desired movements. Synergies can be altered after SCI, for example reflecting patterns of muscle co-activation emerging after the injury	Baniasad (2018); Barroso (2016); Hayes (2014); Perez-Nombela (2017); Zariffa (2012)
Co-contraction index	Commonly obtained by the ratio of agonist to antagonist sEMG amplitude. Changes in supraspinal input and reorganization in the spinal circuitry after SCI can lead to altered patterns of co-activation	Boorman (1996); Cremoux (2017); Gomez-Soriano (2010); Gorassini (2009); Sherwood (1996); Stahl (2015); Tang (1994); Thomas (1998)
Coherence	An index used to indicate functional connections between the cortex and muscles or to indirectly measure shared influence in activating pairs of muscles (intermuscular coherence) or distinct motor pools within the same muscle (intramuscular coherence). After SCI, changes in intramuscular and intermuscular coherence at β or lower frequency bands (< 13 Hz) have been associated with changes in supraspinal inputs and spasms, respectively	Aguiar (2018); Barthélemy (2010); Bravo-Esteban (2014); Bravo-Esteban (2017); Cremoux (2017); Hansen (2005); Norton (2006)
Power spectral density (PSD)	PSD is the frequency domain power distribution of the sEMG. It is often computed using a Fast Fourier Transform. Characteristics of the PSD after SCI can be used to describe phenomena including clonus and fatigue.	Magnusson (1996); Onushko (2007); Steldt (2004); Wang (2013)
Median frequency (MDF)	Median value of the PSD of the sEMG signal, which can contribute to summarizing changes in the power spectrum	Niemeyer (2004); Umezu (2003); Uzun (2012).
Wavelets	Provides a time-frequency decomposition of the sEMG, which can be helpful in analyzing the frequency profiles of multi-stage movements (e.g. gait)	Meyer (2020); Mummidisetty (2012)
Motor unit pattern, number, and size	The number, size, and firing patterns of motor units recorded at the muscle surface. Although limited work exists on motor unit decomposition in sEMG after SCI, findings indicate altered firing patterns, reduced motor unit number, and increased size after SCI	Li (2012b); Li (2015); Winslow (2009); Witt (2020); Xiong (2008); Yang (1990)
Sample entropy	A measurement of the complexity of the sEMG time-series	Liu (2016)
Zero crossings	The number of zero crossings in the sEMG time-series	Corbett (2014)
Fourth order autoregressive coefficients	The coefficients of the fourth order autoregressive model of the sEMG time-series	Lu (2019)
Waveform length	The length of the sEMG time-series	Lu (2019)

#### Time- and frequency-domain characteristics during volitional efforts

A wide variety of signal processing approaches offer opportunities to describe a recorded signal. The resulting metrics can commonly be categorized as time-domain (i.e., metrics derived from the signal represented as a function of time) or frequency-domain (i.e. metrics derived from the signal transformed into a new representation where at least one axis corresponds to frequency). The amplitude metrics discussed above are time-domain metrics, but are dealt with separately in this article because of their widespread use. Other time-domain sEMG properties have been extensively used in the myoelectric control literature, and are thought to provide indirect information on motor unit activation [116, 117]. Common examples include mean absolute value, variance, zero crossings, slope sign changes, waveform length, and Willison amplitude. The mean absolute value is the mean absolute value of signal x(t) in an analysis time window with N samples. Zero crossings is the number of times signal x(t) crosses zero within an analysis window. Slope sign change is related to signal frequency and is defined as the number of times that the slope of the EMG waveform changes sign within an analysis window. Willison amplitude is defined as the number of times that the change in EMG signal amplitude exceeds a threshold; it is an indicator of the firing of motor unit action potentials [116]. Frequency-domain sEMG properties are often associated with central fatigue mechanisms [118–120], but also motor unit activation and brain- or spinal cord-generated rhythms [13, 121–123].

##### Time-domain features during volitional effort

Only a few studies employed these analyses in SCI-related studies. Some studies performed detailed time-domain-based feature extraction to optimize myoelectric pattern recognition-based control systems, using, for example, the mean absolute value, zero crossings, waveform length, slope sign changes, and fourth-order autoregressive coefficients [124–126]. While the high accuracies achieved reinforce that substantial motor control commands can be extracted from partially paralyzed muscles using time-domain features after SCI, this type of study design provides limited insight into the effect of SCI on the sEMG properties. Another time-domain feature, the sample entropy, was also used to develop an algorithm to detect volitional effort onset in SCI, especially due to the spontaneous firing of motor units at rest that contaminates the sEMG signal [127–129].

##### Frequency-domain characteristics and firing frequency during volitional efforts

SCI decreases the motor unit firing during volitional drive. During maximal voluntary effort of the hand thenar muscles, the maximal motor unit firing rate of SCI participants was found to be roughly 15 Hz, much lower compared with 34 Hz in non-injured participants [130, 131]. Although the motor unit activities are reflected in the sEMG signals (using specific electrode configurations—further described in “Amplitude-based sEMG during volitional efforts” section) [132], the motor unit firing rate is often quantified using intramuscular EMG, which is beyond the scope of this review [131]. We focus here on studies analyzing power and frequency of volitional effort using sEMG only.

Changes in sEMG frequency-domain patterns have been associated with improvements in walking; for example, after training, the amount of power in the 7- to 9-Hz clonus band decreased [133]. Variations in muscle activation across gait phases during walking after SCI have been demonstrated using a frequency domain representation based on the Fast Fourier Transform (FFT) [77]. Similarly, wavelet analysis can characterize the time–frequency profile of the sEMG signal with good resolution and contribute to the understanding of targeted walking and corticospinal integrity. Using such techniques, it was shown that an increase of relative semitendinosus intensity in the 38-Hz band during the swing phase was related to targeted walking in SCI, likely reflecting greater corticospinal control before heel-strike during targeted walking [134].

Finally, reduction in mean and median frequencies is a common indicator of increasing levels of muscle fatigue. This reflects that the mechanisms of central fatigue are likely related to the reduced firing of motor units. Early studies in SCI populations took advantage of these properties to test fatigability during wheelchair propulsion and showed how elite wheelchair athletes displayed improved endurance and a slower decline in these frequency-based sEMG properties [118]. Similarly, compared to non-athletes with SCI, individuals with SCI who played wheelchair basketball displayed reduced fatigue [120]. Further studies also reported that spectral analysis of sEMG has been useful clinically to detect muscle fatigue in the context of assistive technology use [119].

##### β-band muscle coherence

β-band activation in cortical motor networks leads to cortical UMN commands often being synchronized within this frequency. As such, β-band frequency is evident in the sEMG properties of intra- and intermuscular coherence.

Norton and Gorassini have shown that changes in cortically related intermuscular coherence are associated with improvements in locomotor skills following treadmill training, likely mediated by increases in the corticospinal drive to muscles [135]. Intramuscular coherence studies indicated that the main characteristics of coupling between tibialis anterior motor unit activity is in the swing phase of AB controls (i.e., peaks of coherence around 10–20 Hz), but are absent or greatly reduced in SCI participants [136]. Interestingly, SCI participants who display foot drop also showed reduced or absent intramuscular coherence of the foot dorsiflexors during walking [137]. Dorsiflexor intramuscular coherence during volitional effort appears to be related to muscle strength and gait function, and may constitute a measure of muscle strength, gait, and spasticity [121], with the ability to indicate longitudinal adaptive and maladaptive motor control mechanisms [138].

Frequency-based sEMG analysis may also be used to investigate corticomuscular coherence following SCI using electroencephalography. Corticomuscular coherence at lower frequencies (≈ 10 Hz) may indicate decreased cortical influence on spinal centers, leading to increased muscle co-activation [123]. Interestingly, spasticity was also associated with lower dorsiflexor intramuscular coherence in the 15–30 Hz band during volitional effort, suggesting that lower coherence is associated with the spontaneous firing of LMN [138]. This will be discussed further in “Time- and frequency-domain characteristics at rest” section.

### Properties reflecting LMN spontaneous activity

SCI reduces the UMN input to the spinal cord circuitry and creates an opportunity for plasticity and reorganization. Synaptic territory may be invaded by preserved connections not interrupted by the SCI, afferents arriving from the periphery (e.g., mechanoreceptors and proprioceptors), and/or pathways within the intrinsic spinal cord circuitry. As a consequence, the temporal and spatial summations of action potentials become unbalanced in the intrinsic spinal cord circuitry and more prone to excitation from the periphery, alongside the reduction of cortical inhibition. This leads to the emergence of hyperreflexia and long-lasting spontaneous firing of LMNs, and also suggests the loss of inhibitory interneurons within the spinal cord circuitry [139]. Amplitude-based sEMG properties can capture the occurrence of this involuntary activity at rest, namely muscle spasms. Muscle spasms are often characterized as unit, clonus, or tonic spasms. In this section, we summarize the available evidence on how the sEMG signal can reflect spontaneous firing in the LMN.

#### Amplitude-based sEMG at rest

Harnessing the residual brain influence on spinal cord function includes increasing volitional activation but also reducing involuntary spontaneous activity. sEMG activity below the lesion can be studied in great detail using sEMG amplitude-based properties at rest. For example, similar to volitional contractions but tailored to quantify the response of paralyzed muscles to tendon taps, vibration, and plantar stimulation, the Brain Motor Control Assessment score can reflect the presence or absence of reflexes or the ability of voluntary suppression [40]. The results indicated that 84% of SCI participants with clinically motor complete SCI were able to demonstrate at least one of the defining features of residual, subclinical brain influence, and could be categorized as ‘discomplete’ SCI [40]. Data also support the Brain Motor Control Assessment as highly comparable between sessions when patients are stable [39], indicating the possibility of conducting extensive assessments throughout the recovery process. The Brain Motor Control Assessment was also applied to the upper limbs of SCI participants and a significant level of involuntary muscle activity at rest was found compared to AB control participants. These involuntary activations reduced over time (from ≈ 86 to ≈ 500 days post-SCI) and became similar to AB control participants [43].

Early reports of involuntary sEMG activity in SCI described how the deprivation of supraspinal control leads to an increase in LMN activities. This was manifested in sEMG response induced by passive knee movements, as sinusoidal torques can occasionally induced phasic sEMG and spasms in individuals with SCI [140]. The existence of these abnormal responses was further investigated by testing and modelling spasticity [141, 142]. Later it was demonstrated that voluntary supraspinal suppression of spinal reflex activity was possible for some SCI participants [143, 144]. Following these initial studies of reflex activity, later studies provided more information on how mechanoreceptors, proprioceptors, and the intrinsic spinal cord circuitry mediate the spontaneous activity of LMN and hyperreflexia [98, 145–151] (refer to Additional file 1: Figure S1 for a summary of additional instrumentation used in the studies reviewed).

Reflex responses in the presence of patterned stimuli including rhythmic movements and vibrations have been investigated on multiple occasions [91, 93, 96, 131, 149, 152–162]. These investigations have described relationships between spastic reflexes, afferent input patterns and multi-joint responses, and provided insights into the underlying neural circuitry.

Some motor units may show prolonged, contraction-induced firing after the voluntary contraction. This induced motor unit firing may last for minutes—also denominated as unit spasms [130]. The examination of the recruitment, firing rate modulation, and de-recruitment of motor units that underlie spasms of thenar muscles in SCI indicated that mean sEMG and force were strongly associated during the spasms and that some motor units were not de-recruited following spasms but rather continued to fire for several minutes at low firing rates [163]. sEMG provides objective measurements of naturally occurring spasms in contrast to the self-reported spasm counts, which are often used to make clinical decisions, but the former usually involves visual inspection of long sEMG recording [164]. The automatic identification of spasmodic events in long-term sEMG recordings enabled further understanding of these phenomena [165, 166]. It is feasible to detect spasms since they typically involve a rapid rise in sEMG amplitude followed by a more gradual fall, which is also observed in torque. Interestingly, it was later shown that individuals with SCI may adaptively use spasms to increase force production [131].

Multiple types of sEMG analyses were used to link muscle activity and spasticity measured through the Ashworth scale. Some positive correlations were found by linking sEMG amplitude-based and timing properties, e.g. mean, peak, and the time between onset and peak of electrical activity [167]; predicting the level of spasticity using sEMG features (i.e., RMS of five muscles under two maneuvers) and machine learning algorithms [168]; and, using amplitude-based sEMG data from the Brain Motor Control Assessment [41] and the spinal cord assessment tool for spastic reflexes [169]. The assessment of spasticity using full range passive movements indicated sEMG RMS increased with increasing stretch velocities, providing an objective outcome [170].

Subsequent studies reported weak correlations between the Ashworth scores and reflex activity, and a unique reflex mechanism in SCI was proposed [171]. Similarly, there was a weak association between long-term sEMG recordings (i.e. number and duration of burst) during ADLs and self-reported level of spasticity [172]. The subjective component and lack of reliability in reporting spasticity using the Ashworth scale have been pinpointed as a possible confounding factor in establishing a clear relation between sEMG properties and spasticity [173]. However, the use of sEMG activity was recommended to investigate reflex hyperexcitability and to determine the occurrence of muscle spasms [173]. For example, sEMG measurements of involuntary activity in the lower extremity were not significantly related to perceived impact of spasticity on daily life, although spasm duration was positively associated with clinical extensor spasticity [174]. These studies suggest that the quantification of sEMG during involuntary contractions is important to better understand the relation between neurophysiological and self-reported measures of spasticity. A possible explanation to these contradictory findings and recommendations may lie in the fact that individuals with SCI describe spasticity in terms of their spasms and tone. Thus, multiple tests may be necessary to fully capture both the biological basis and functional impact of spasticity [175].

#### Time- and frequency-domain characteristics at rest

Studies on the time-domain sEMG properties at rest are lacking in SCI, which is understandable since time-domain features were first proposed for the active myoelectric control of upper-extremity prosthetic devices [116, 117].

Frequency-domain sEMG properties are highly susceptible to noise given the low signal power in sEMG recordings at rest. For example, the investigation of sEMG activity during passive static stretch showed characteristics of “white noise” in the power density spectrums at rest with a median frequency of 400–548 Hz (AB controls) and 478–540 Hz (SCI) without a characteristic concentration of frequencies in the spectrum, and with strikingly low power [176].

##### Frequency-domain characteristics and firing frequency of spontaneous motor unit firing

sEMG analysis at rest may provide information on the rate of involuntary motor unit firing and on whether medications can dampen such activity. Few methods have been proposed to identify spontaneous motor unit firing in SCI using sEMG. An automated multi-step classification algorithm of sEMG from paralyzed thenar muscles enabled the detection and classification of spontaneously firing motor units using sEMG [132]. The motor unit number index, another method for estimating the motor unit size and number, was also shown to capture the difference between intact muscles and those paralyzed in SCI—reduced number and greater size of motor units in SCI [26, 177–180]. The main difference between these two methods is the use of a specific configuration of surface electrodes [132], in contrast to the use of standard bipolar surface electrodes configuration (but with the addition of a normalization step using electrical stimulation) [177]. Despite the important applicability in SCI, these methodologies were not broadly employed, and as mentioned earlier, most of the studies included in this scoping review assess firing rates using a combination of sEMG and intramuscular EMG (similar to “Frequency-Domain Characteristics and Firing Frequency During Volitional Efforts” Frequency domain and firing frequency during volitional efforts). For instance, the seminal work of Thomas, Zijdewind, and colleagues broadly used the above-mentioned combined approach to understand motor control of the hand thenar muscles in SCI [181].

The involuntary recruitment of populations of motor units during clonus has been described in detail in SCI. It was shown that the clonus frequency ranged from 4.7 to 7.0 Hz and the firing of motor units seemed to follow orderly recruitment during these involuntary contractions. This pattern of recruitment is consistent with motor unit recruitment seen during many voluntary contractions, and suggests the importance of spinal mechanisms in the control of motor unit behavior in SCI [182]. Another nuance of spontaneous motor unit firing in the hand is the regularity of firing observed in some motor units. Regularly firing motor units seem to be more excitable as they displayed longer after hyperpolarization potentials and higher mean firing rates, likely reflecting active properties (such as persistent currents) within motoneurons. This activity is seen in the absence of voluntary drive but also may underlie the firing patterns typically recorded during voluntary contractions [183]. Indeed, it was shown that coactive motoneurons are likely driven by synaptic inputs from different sources during muscle spasms [163].

##### Low-frequency muscle coherence

In contrast to the β-band synchronization of UMN signals to the spinal cord, the intrinsic spinal cord circuitry is thought to operate at lower frequencies. Lower frequency oscillation seems to be associated with spasticity during volitional contractions [138] but also with spasms [13]. The recording of sEMG during a 24-h period using a wearable device allowed the detection of natural spasms and the calculation of intermuscular coherence in a set of lower limb muscles. Intermuscular coherence during the spasms occurred at low frequencies (between 2 and 13 Hz) in complete SCI, but at higher frequencies in incomplete SCI. The current evidence suggests that the most likely source for this low-frequency coherence is the spinal cord and its peripheral feedback loops, given the different responses depending on the lesion profile [13].

## Discussion

This scoping review aimed to examine the properties of the sEMG following SCI. Given the increasing role of technology and the possibility to assess neurorecovery in more detail, this review aims to support the interpretation of sEMG signals after SCI, facilitate the choice of sEMG methodology for planning and conducting research in SCI, support the development of assistive technologies, as well as highlight gaps in knowledge.

Among the identified body of evidence, the minority of studies included sEMG measurements beyond amplitude-based analysis. Most studies employed amplitude-based analysis using RMS (57 studies), normalized sEMG (e.g. %EMG, %MVC, and %peak; 33 studies), and qualitative or semi-quantitative scores of the sEMG pattern (16 studies). In contrast, only 19 studies used time-domain or frequency-domain analysis (Table 1). The summarized findings from this review suggest that amplitude-based analysis is effective in indicating muscle strength and recovery following SCI, including important aspects of multi-muscle coordination. Despite these positive findings, time- and frequency-domain analysis may describe the sEMG properties in ways that are not possible with amplitude-based analysis alone, providing a more detailed description of the neurophysiological changes following SCI. Time- and frequency-domain sEMG properties are thought to reflect the motor unit firing patterns, either spontaneous or cortically driven, but more studies are needed to consolidate the relation of these sEMG properties with physiological events. Ultimately, sEMG signals may be better characterized if a broader range of properties is considered.

**Fig. 7:**
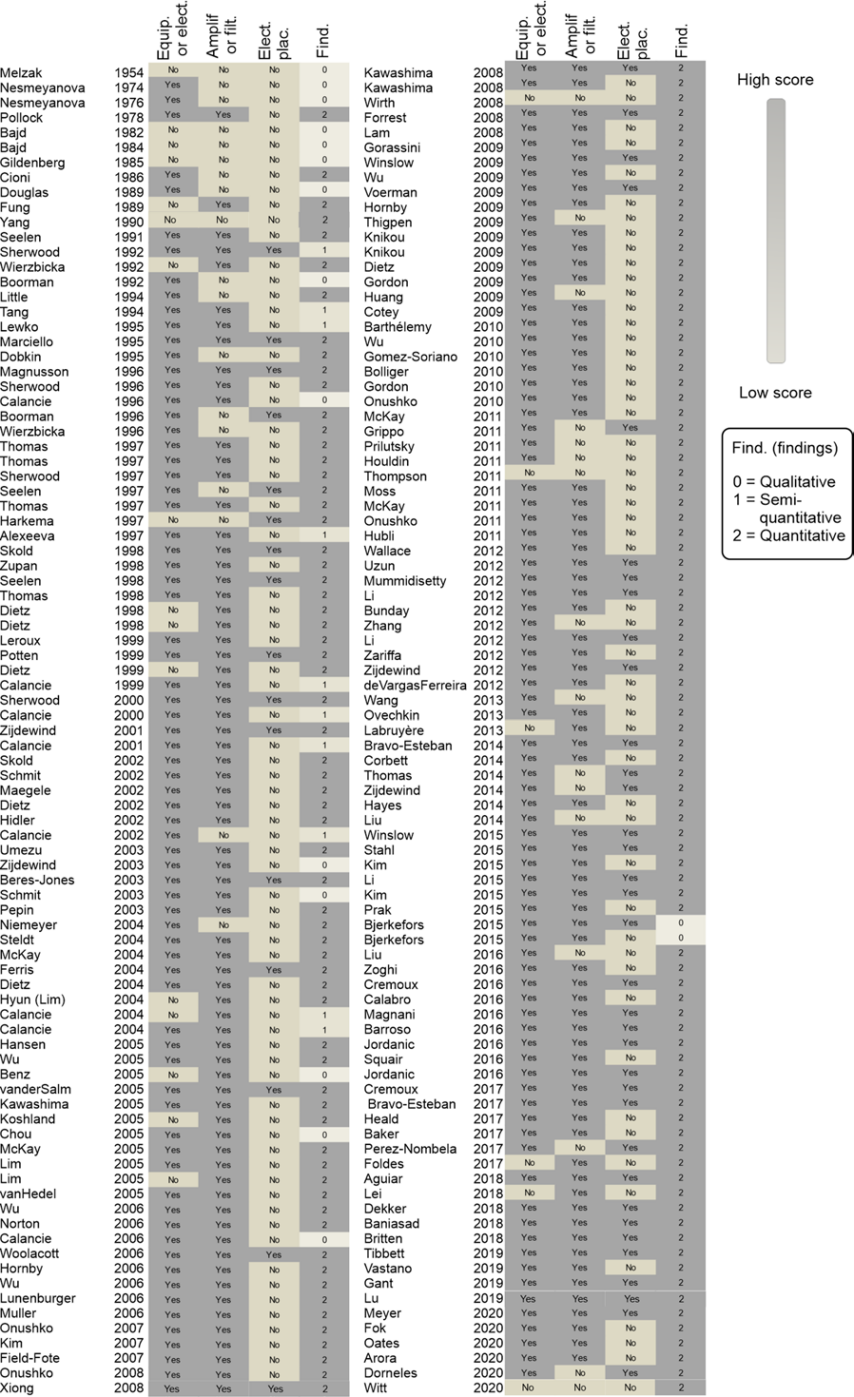
Description of the surface electromyography (sEMG) methodology. The presence or absence of the description of the sEMG equipment or electrodes, the amplification or filtering procedures, the detailed placement of electrodes (or proper citations) and the type of findings (qualitative: based on visual sEMG graph analysis; semi-quantitative: based on scores of the sEMG pattern; quantitative). *Equip.* sEMG equipment, *Elect*. sEMG electrode, *Filt*. sEMG hardware or digital filtering, *Plac*. electrode placement on muscles, *Find*. sEMG outcomes, *FFT* Fast Fourier transform, *PSD* Power Spectral Density, *MDF* median frequency, *RMS* root mean square

An important methodological consideration when dealing with amplitude-based sEMG properties is their susceptibility to inter-day and inter-subject variability due to variations in factors including electrode locations and skin impedance. Although normalization (e.g., to MVC) is widely used to attempt to compensate for these issues, a limitation of most sEMG studies in SCI was the lack of reported electrode placement and between-days reliability of the sEMG measurements. Only 51/178 references reported detailed information about electrode placement, but the majority reported details about the sEMG equipment or filtering/amplification procedures (Fig. 7); and 57% of studies used an AB control group. Although the use of an uninjured control group depends on the research question, appropriate experimental controls are highly necessary to deal with the stochastic and variable nature of the sEMG signal after SCI. Finally, the well-powered studies from Calancie and Sherwood in the 90’s and 2000’s are notable but employed mostly semi-quantitative sEMG analysis [10, 30, 31, 40, 41, 168].

Another consideration is the relatively small number of studies combining intramuscular EMG and sEMG methodologies. Simultaneous recordings using the two techniques have the potential to improve our understanding of how motor unit firing patterns are reflected in the sEMG. The importance of this scoping review is supported by a recent systematic review on the electrophysiological outcome measures in SCI clinical trials, which indicated 27/64 of clinical trials used sEMG analysis and only 1/64 used intramuscular EMG [184]. It is reasonable to view sEMG as the primary choice of neurophysiological method in SCI clinical trials, given its non-invasiveness and ease-of-use. Recent improvements in motor unit decomposition from sEMG offer an exciting opportunity to directly measure motor unit firing rates and obtain greater neurophysiological insights without the need for invasive measurements [185, 186]. However, these methods require specialized instrumentation, and their use in SCI research has been limited to date.

A broader perspective on the sEMG signal characteristics has the potential to lead to new outcome measures for use in clinical trials, and to benefit the field through the knowledge gained in understanding the relation of these properties with physiology. Most of the studies exploring time- and frequency-domain sEMG properties in detail are conducted in non-injured subjects, other populations (e.g. amputees), or with an insufficient number of SCI participants (less than 4 SCI) to be included in this scoping review. On the other hand, the SCI studies with larger samples mostly rely only on amplitude-based analysis. This creates an inverse relationship between the sample size and the variety of sEMG properties reported (e.g., properties beyond the amplitude).

In the new era of machine learning, the characterization of the volitional sEMG activity below the lesion has a potential application as a screening tool in the clinical settings [14, 187]. Advancement in technology may promote the development of novel devices with increased portability and ease-of-use. Clinicians may use these sEMG tools during neurorehabilitation, with potential implications to diagnosis and to the optimization of treatment time. Additional characterization of sEMG after SCI may also support the development of assistive technologies such as myoelectric control interfaces [22, 117, 124].

### Implications for clinical practice

The utility of sEMG is widely appreciated as it is seen to provide simple and easy-to-use assessments of motor impairments and rehabilitation after SCI. On the other hand, as recently described by Pilkar et al*.* and Merletti et al*.*, there is a range of factors to consider when implementing sEMG assessments in clinical practice [188, 189]. For instance, sEMG requires dedicated resources and infrastructure for equipment, training, and maintenance. Health professionals will need specialized training, ongoing support, and easy-to-use sEMG interfaces [189]. In this context, this scoping review has identified a body of consistent evidence indicating that sEMG is an informative complement to current clinical testing (e.g., MMT), but likely not being fully utilized in terms of the information that it can provide. Looking forward, engineers and software developers must develop sEMG systems that make a wider range of metrics available at the point of care, not restricted to amplitude-based calculations. For example, if easy-to-use sEMG information is available preoperatively, it may avoid common pitfalls in selecting potential donor and recipient muscles when attempting surgical nerve transfer to restore upper limb function in SCI [190]. More broadly, a comprehensive understanding of the spontaneous recovery profile in sEMG may provide valuable guidance for therapy selection and progression.

### Implications for research

From a research perspective, the availability of high-quality outcome measures is essential to the successful translation of new interventions. Recent reviews by Hubli et al*.* and Korupolu et al*.* have described the use and benefits of electrophysiological outcome measures, including sEMG, in the context of SCI clinical trials [7, 184]. Remaining avenues for improvement include greater standardization as well as ease of implementation. Deeper characterization of the sEMG signal can play a role in this context by identifying signal properties that have suitable psychometric properties to be incorporated into outcome assessments, as well as by improving our understanding of how different metrics relate to the underlying physiology. The potential also exists to simplify data collection, if signal processing or machine learning can be used to extract subtle trends from non-invasive data during simple protocols, rather than requiring invasive techniques or complex stimulation protocols. For instance, the adoption of methods to estimate motor unit firing using sEMG should further advance the understanding of sEMG properties in SCI. The present review lays the groundwork towards these goals.

### Limitations

Our study had some limitations concerning the exclusion of studies on treatment or interventions and the lack of studies encompassing recent advances in high-density sEMG. First, given our interest in how the sEMG properties are changed in response to an SCI, it was considered that any intervention or treatment would have the potential to interfere with the signal characteristics. For example, the use of epidural or transcutaneous spinal cord stimulation may generate electrically induced muscle activation that interferes with the understanding of how the sEMG is changed after an SCI. Other types of treatments were identified, such as pharmacological or rehabilitative (e.g., locomotor training, EMG biofeedback). The most common interventions were the use of assistive devices (e.g., robot-assisted training, exoskeletons, wheelchair). Although the review of these studies would be important, they are outside the scope of our present question and we suggest that future reviews should provide guidance on appropriate methods to use sEMG to answer specific research and clinical questions (including interventional trials).

**Fig. 8:**
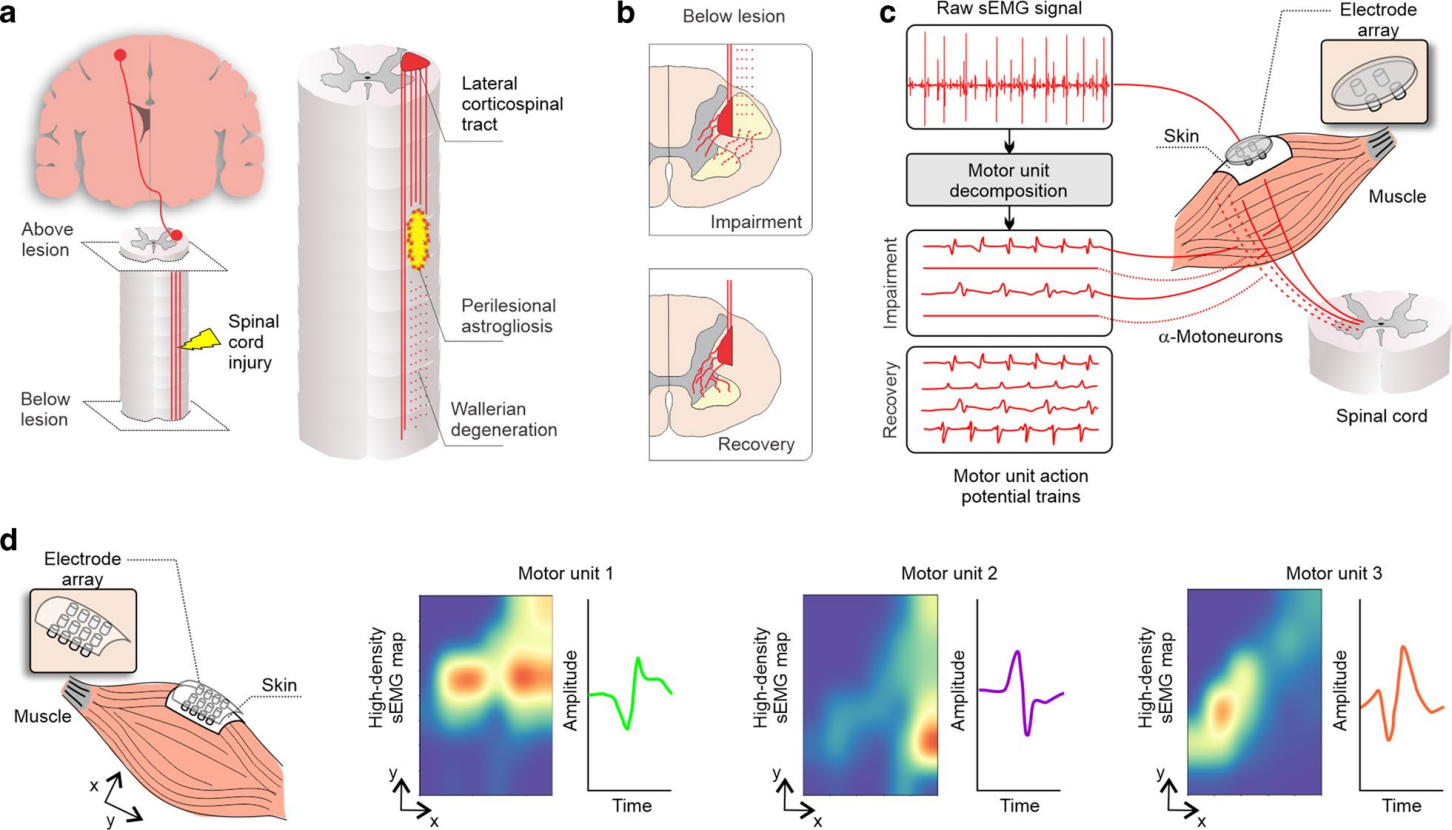
Overview of conceptual work on high-density sEMG, motor unit decomposition, and its application in SCI. **a, left** Corticospinal projections (red) and spinal cord injury (yellow). **a, right** In the intermediate and chronic phases (2 weeks to 6 months), axons continue to degenerate and the astroglial scar matures to become a potent inhibitor of regeneration (restrict axonal regrowth and cell migration). The lateral corticospinal tract (red) is the major descending motor tract, which may be damaged after SCI (red hashed lines) [192]. **b, upper panel** The remaining projections from the corticospinal tract (red solid lines) synapse with α-motoneurons in the spinal cord to control volitional movements—(**b, lower panel**) which undergo extensive plasticity with motor recovery [193]. **c** Standard sEMG is able to capture the overall activity of these motor units but four or five small pin electrode arrays are able to decompose the raw sEMG signal into individual motor unit potential trains [186, 194, 195], with potential to track the impairment and recovery of motor unit control after an SCI. The most common techniques used for motor unit decomposition involve the use of the progressive FastICA peel-off framework [196–198], multichannel blind source separation using convolution kernel compensation [199–201] or specific algorithms, e.g., using machine-learning and time-varying shape discrimination [186, 194, 195]. **d** The use of multi-electrode arrays increases the spatial resolution; in addition to the motor unit decomposition, multi-electrode arrays can also unveil the territory of each motor unit [202]

Secondly, our review identified a lack of studies on high-density sEMG and motor unit decomposition in SCI. Studies on high-density sEMG were mostly conducted in AB participants or in individuals with an SCI but with insufficient sample size to be included in the present review. We believe that the identification of this knowledge gap is an important finding of this scoping review and should further well-sampled and comprehensive studies on how these novel techniques can be applied in SCI. Recently, novel methodological advancements in high-density sEMG preprocessing have been proposed to enhance the diagnostic power in individuals with an SCI, which will help to develop a standard sEMG preprocessing pipeline [191]. In Fig. 8, we provide a simplified overview of some of the conceptual work on high-density sEMG and motor unit decomposition and provide our perspective on how the SCI field can take advantage of these techniques.

An additional limitation is the focus here on limb and trunk muscles. The inclusion of respiration and sphincter muscles would warrant further work, as the differences in neural control and muscle properties could be expected to alter the sEMG properties.

## Conclusion

The research on sEMG in SCI over the past seven decades has accumulated abundant evidence about changes in the sEMG properties after the injury. Most of the studies describe muscle weakness, coordination and spontaneous activity using sEMG amplitude properties. It is known that sEMG can capture the residual motor command in great detail, including in muscles below the level of injury with seemingly absent motor activities. Therapies promoting sensorimotor recovery aim at harnessing these residual supraspinal inputs to increase muscle strength and coordination while reducing spontaneous activity. Thus, the inclusion of sEMG assessments in the clinical setting affords important information on how novel therapies may engage and optimize the residual motor command. Nonetheless, current gaps include the lack of studies reporting changes in sEMG properties beyond the amplitude measurement. In order to advance the field, we suggest the incorporation of a broader range of signal properties into the neurophysiological assessment post-SCI and the development of a greater understanding of the relation between these sEMG properties and underlying physiology.

## Supplementary Information

**Additional file 1: Figure S1. **Additional instrumentation used in all studies (a), studies assessing sEMG properties at volitional effort (b) and rest (c). **Table S1.** Search strategy.

## Data Availability

Not applicable.
