# Supplementary material for: Properties of the surface electromyogram following traumatic spinal cord injury: a scoping review
Source: J Neuroeng Rehabil. 2021 Jun 29;18:105. doi: 10.1186/s12984-021-00888-2 (PMC8244234; doi:10.1186/s12984-021-00888-2)
Supplement: Supplementary file 1 — Additional file 1: Figure S1. Additional instrumentation used in all studies (a), studies assessing sEMG properties at volitional effort (b) and rest (c). Table S1. Search strategy. [file 12984_2021_888_MOESM1_ESM.docx]

**Figure S1**

**
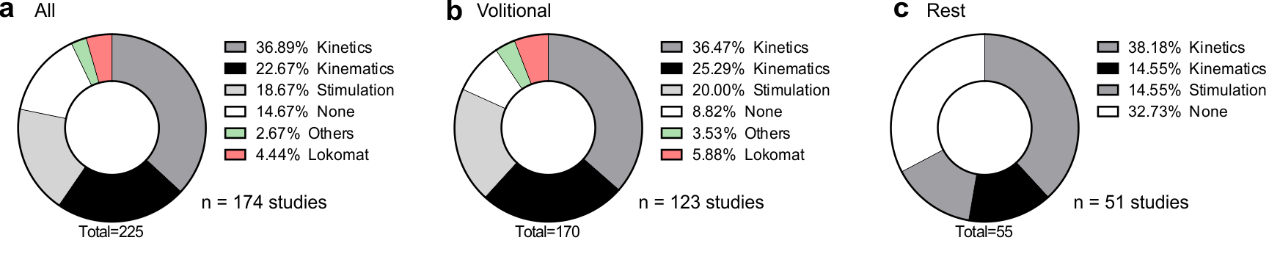
**

**Figure S1.** Additional instrumentation used in all studies (**a**), studies assessing sEMG properties at volitional effort (**b**) and rest (**c**).

**Table S1**

**Search Strategy: Ovid MEDLINE(R) ALL <1946 to September 22nd, 2020>**

--------------------------------------------------------------------------------

**1 [Population: Spinal Cord Injury]**

2 exp Spinal Cord Injuries/

3 exp Paraplegia/

4 exp Quadriplegia/

5 (spinal cord adj3 (injur* or contusion* or trauma* or transection* or laceration* or compression* or lesion*)).tw,kw.

6 (paraplegi* or quadriplegi* or tetraplegi*).tw,kw.

7 (myelopath* adj2 (traumatic or post-traumatic or post traumatic or compressive)).tw,kw.

8 or/2-7

**9 [Concept 2: EMG]**

10 Electromyography/

11 (electric* myogram* or electromyogra* or electro myogra* or EMG or polyelectromyogra*).tw,kw.

12 10 or 11

**13 [Concept 3: Muscle/Motor Response]**

14 exp Motor Neurons/

15 exp Muscle Contraction/

16 Muscle Spasticity/

17 exp Muscles

18 (motorneuron* or motoneuron* or neuromotor* or motorunit*).tw,kw.

19 (motor* adj3 (nerve* or neuron* or unit* or subunit* or control* or task* or function*)).tw,kw.

20 (motor unit* adj3 (recruitment* or derecruitment* or fired or firing or activ* or propert* or behaviour* or behavior*)).tw,kw.

21 ((muscle* or muscular*) adj3 (contract* or activ* or action* or reaction* or spasm* or respon* or tone or demand* or spastic*)).tw,kw.

22 (inotropism* or myocontraction* or intermuscular coherence).tw,kw.

23 or/14-22

24 8 and 12 and 23

25 limit 24 to "humans only (removes records about animals)"

***************************
